# Investigating the spatial variations of high prevalences of severe malnutrition among children in Papua New Guinea: results from geoadditive models

**DOI:** 10.1186/1756-0500-5-228

**Published:** 2012-05-11

**Authors:** Handan Wand, Namarola Lote, Irene Semos, Peter Siba

**Affiliations:** 1 The Kirby Institute, University of New South Wales, Sydney, Australia; 2 Papua New Guinea Institute of Medical Research, Goroka, Papua New Guinea

**Keywords:** Malnutrition, Stunting, Wasting, Children, Papua New Guinea

## Abstract

**Background:**

Papua New Guinea (PNG) is one of the nutritionally vulnerable countries with a high rate of children death without showing a sign of improvement in last two decades. Current study investigated the prevalences of stunting and wasting among a cohort of children in PNG and described the spatial features of these outcomes at the province and district-levels.

**Objective:**

To determine the prevalences of stunting and wasting among a cohort of children in PNG and to describe the spatial features of these outcomes at the province and district-levels. We also described the spatial features of these outcomes at province and district-levels.

**Methods:**

The health and nutritional status of 683 children aged less than five years was assessed using a cross-sectional multi-stage household survey conducted in the Eastern Highlands and Madang Provinces of PNG during the period of 2003–2004. Growth z-scores such as height-for-age and weight-for-age were generated using World Health Organization classifications.

**Results:**

The prevalences of stunting (height-for-age z-score less than −2.0) were 59% and 49% in the Eastern Highlands and Madang respectively (*P* = 0.019). The prevalences of wasting (weight-for-height z-score less than −2.0) were 14% and 22% in Eastern Highlands and Madang respectively, (*P* = 0.039); overall, only 21% of the children had completed all their scheduled vaccines and 95% of the caregivers had less than primary school education. Our statistical maps showed considerable spatial variations (province- and district-levels) with regard to the stunting, wasting and other key factors within a relatively small geographical region.

**Conclusions:**

Current study determined one of the highest prevalence of stunting among children in PNG. The impact of geographical locations on the risk factors must be recognized as it affects epidemiology and intervention coverage.

## Background

Childhood malnutrition is a serious risk factor for poor health and is known to be associated with increased risk of impaired cognitive development and lower educational achievement [1,2]. Despite worldwide prevention efforts, approximately 150 million children continue to suffer from malnutrition worldwide [3,4]. Proportion of anthropometric indices “height-for-age z-score < −2”, “weight-for-height z-score < −2” and “weight-for-age z-score < −2” are commonly used measures to determine the severe forms of malnutrition such as stunting, wasting and underweight respectively. In developing countries it is estimated that approximately one quarter of the children less than five years of age are growth-retarded which contribute substantially to the burden of diseases, in developing countries [5]. Therefore, understanding the prevalence and patterns of malnutrition, particularly stunting and wasting in children, is of critical importance for public health policy.

Papua New Guinea (PNG) is located in the south-western Pacific Ocean, and is one of the most culturally diverse countries in the world with over 850 native languages. Out of a population of just over 5 million 85% live in rural areas [6]. However, PNG has also one of the highest under-five child mortality rates in the world. The 2000 national census reported the underage five mortality rate as 94 per 1000 live births and infant mortality rate as 70 per 1000 [7]. PNG was named by UNICEF to be one of the four countries in the world for which the child mortality rate has not showed any decline since 1980 [7,8]. Reports on child health in rural areas of PNG indicate high prevalence of diseases such as pneumonia, measles, meningitis and malaria causing high rates of child mortality. Overall, vaccination coverage was estimated to be 64% for diphtheria, tetanus, pertussis and 60% for measles, while, in the Western Province, these estimates were only 30% and 27%, respectively; in Eastern Highlands Province in 1997, coverage was about 33% for immunizing doses of any vaccine [6-8]. Urgent action needed to ensure that effective interventions are integrated into programmes that aim to improve child health outcomes in PNG, particularly in rural areas.

In this paper, geoadditive models, an extension of generalized additive models, were used to describe the spatial variations of severe forms of childhood malnutrition such as stunting, wasting and underweight using the data from the household survey. At a geographical level, poverty, health services, cultural differences, ethnic norms and practices are some of the factors which cannot be measured or captured easily. The rationale is that a spatial effect is usually considered to be a surrogate of these unobserved/unmeasured environmental influences which can also affect the consequences of severe malnutrition such as stunting and wasting. Identifying spatial patterns of severe malnutrition will also assist in poverty mapping and associated local district-level variations for these adverse health outcomes among children.

This is the first study to investigate the links between child’s health as determined by the level of malnutrition and geographic location in provinces of PNG using modern statistical techniques. Our analysis serves as an example of how the spatial variations in selected covariates can be detected within relatively homogeneous areas using data from the Household and Community Practices Survey, rather than conducting a complete investigation of the geographical variation and risk factors of the severe childhood malnutrition in this region.

## Methods

### Study population

The World Health Organisation (WHO) initiated the Integrated Management of Childhood Illnesses (IMCI) to inform to the developing world of issues related to the severity of deaths in children under 5 years of age. But, most importantly, the programme developed a new and effective child health strategy. The IMCI concept is based on: (1) improvement of management skills of health workers; (2) improvement of health care delivery systems; (3) growth and development. The last component is specifically related to the Household and Community Practices Survey (HCPS) which shifts the focus of the IMCI from improving the health system to improving household and community practices. The Papua New Guinea Institute of Medical Research (PNGIMR) along with the National Department of Health (NDoH), WHO and funded by the United Nations Children’s Fund (UNICEF) were tasked to carry out the HCPS in peri-urban and rural areas of the Eastern Highlands and Madang Provinces.

A structured household questionnaire was used for collecting information about the child’s survival and health as well as information regarding the mother, father and caregiver. On health, age (months); gender, immunization completed, provision of nutritional status information. In addition caregiver level characteristics such as age and level of education were collected.

The *z*-scores for weight and height were calculated based on the child’s age and gender. The WHO Global Database on Child Growth and Malnutrition recommends a cut off *z* score of *< −*2 to classify low height-for-age (stunting), low weight-for-height (wasting) and low weight-for-age (underweight) as moderate and a *z-*score of *< −*3 to define severe under nutrition [9]. A *z* score of *< −*2 indicates that a child’s height-for-age, weight-for-height or weight-for-age is 2 standard deviations below the age and gender-specific median for the normal population.

The current study only included children with parents who gave written informed consent. The study received ethical approval from the Institute of Medical Research (IMR) Institutional Review Board and the Medical Research Advisory Committee.

### Geographical data

Details of the children’s place of residence were collected during the survey and transformed into longitude and latitude. Each child’s confidentiality was ensured, using identifying numbers linked to coordinates instead of names and addresses. The longitudinal and latitudinal geographical co-ordinates were entered as raw data. The current study included only those children whose place of residence was recorded.

### Geoadditive models

We estimated local disease risks using geoadditive models, an extension of generalized additive models [10]. For example, in an age adjusted analysis, log odds of stunting was modelled as a bivariate function of geographical location, a potential surrogate for unobserved covariates, using bivariate smooth functions $f_{1}$ and $f_{2}$ of spatial coordinates (i.e. *longitude* and *latitude) and* child’s age (months):

(1) $$\text{logit}[P(stunting)=1]=f_{1}{\left(latitute,longitude\right)}_{i}+f_{2}{\left(child's\;age\right)}_{i}$$

Based on this methodology, an image plot of the stunting cases was created as a bivariate function of longitude and latitude. Spatial components of the model i.e. $f_{1}{\left(latitute,longitude\right)}_{i}$ assumed to characterize correlations among the stunting cases.

In a separate analysis, semiparametric logistic regression models were fitted to assess the relationship between the binary outcome of interests such as “stunting” and a continuous covariate such as child’s age and care giver’s age:

(2) $$\text{logit}[P(stunting)=1]=f{\left(child's\;age\right)}^{i}$$

(3) $$\text{logit }[P(stunting=1]=f{\left(caregiver'\text{s age}\right)}^{i}$$

In addition to the visual presentation of the fitted curves, estimated degrees of freedom along with the second derivative plots were used to assess the strength of the association between the outcome variable and covariates of interest (data not shown).

We also investigated the spatial variations of six modifiable risk factors in our analysis: highest level of education that child’s caregiver completed (no education versus at least primary school), immunization status (all completed versus not), breast-feeding (ever versusnever), and one of the following conditions two weeks prior to the survey: cough (including chesty, blocked nose) (yes/no), diarrhea (yes/no). For each factor, a binary variable was created: child received 1 if he/she was in high risk category (i.e. no education, incomplete immunization, never breast fed and having cough or diarrhea two weeks prior to the survey) and 0 otherwise. The risk points were summed across the six modifiable risk factors to obtain a risk score.

Geoadditive and Semiparametric regression models in this study were fitted by using the Semipar package in the statistical software system R (version 2.13.0). All other analyses used Stata 10.0 (College Station, TX) statistical software.

## Results

**Figure 1:**
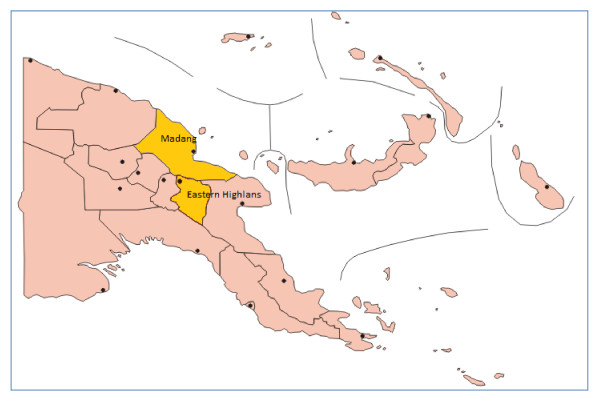
Regions targeted in this study.

The Eastern Highlands Province is located in the highlands of Papua New Guinea (Figure 1). Madang Province is located on the northern coast of mainland Papua New Guinea. Both provinces share a common administrative boundary. Of the 683 children and their 636 caregivers who participated in this study 49% were from the Eastern Highland Province and 51% from Madang Province.

Characteristics of the children are summarized in Table 1. Overall, the median age of the children was 19 months (10–32), 53% of them were male and only 21% of the children have completed all three vaccines (oral polio vaccine, measles and Bacille Calmette-Guérin). More than half of the children were determined to be stunted, approximately 20% of them were wasted and one third of them were classified as underweight. Overall, 43% of the children had at least one of the following conditions in the two weeks prior to the survey: diarrhea, cough (including chesty cough and cough with blocked nose); majority of the caregivers had only primary school education (51%) or no education (44%).

**Table 1:** Demographic characteristics of children and caregivers by Province

**Children**	**Overall** **%**	**Eastern Highlands ** **Province** **%**	**Madang ** **Province** **%**	***p*-value**
All	683	49%	51%	
Age (months), median (IQR)	19 (10–32)	17 (7–32)	21 (11–31)	0.0541
Male	53%	51%	55%	0.439
**Malnutrition characteristics**				
Stunting	54%	59%	49%	0.019
Under weight	31%	29%	33%	0.236
Wasting	18%	14%	22%	0.030
**Immunization completed**^1^				
BCG	80%	80%	80%	0.981
OPV	29%	31%	28%	0.341
Measles	38%	33%	42%	0.019
All 3 vaccine completed	21%	21%	22%	0.650
Breast feeding (ever)	95%	94%	95%	0.400
**Conditions of child**				
Sick	43%	49%	39%	0.015
Diarrhea	17%	23%	12%	<0.001
Cough	55%	59%	52%	0.101
Chesty cough	44%	47%	41%	0.164
**Care givers**				
All	636	49%	51%	
Age (years), median (IQR)	26 (23–33)	26 (22–32)	27 (24–34)	0.423
**Education**				<0.001
No education	44%	52%	36%	
Primary school	51%	43%	58%	
Secondary school	5%	5%	6%	

^1^ Bacille Calmette-Guérin (BCG), Oral Polio Vaccine (OPV), Measles.

Children were broadly similar in terms of age (*P* = 0.0541), gender (*P* = 0.439) and completion rate of all three immunization (*P* = 0.650) when they were compared between the two provinces. However, the prevalence of stunting was significantly higher among children who lived in Eastern Highlands Province compared to those lived in Madang Province (59% versus 49%, *P* = 0.019) while prevalence of wasting was significantly lower in the Eastern Highlands compared to the Madang Province (14% versus 22%, *P* = 0.030). The proportion of children who were reported to be sick (diarrhea, chesty/block nose cough) two weeks prior to the survey was also significant higher in the Eastern Highlands Province compared to the Madang Province (49% versus 39%, *P* = 0.015); more than fifty percent of the children were reported to have had a cough in both provinces (*P* = 0.101); diarrhea was significantly higher among children who participated to the study from Eastern Highlands Province (23% versus 12%, *P* < 0.001). Significantly higher proportion of caregivers from Eastern Highlands Province also reported having no education compared to those from Madang Province (52% versus 36%, *P* < 0.001).

### Spatial analysis

**Figure 2:**
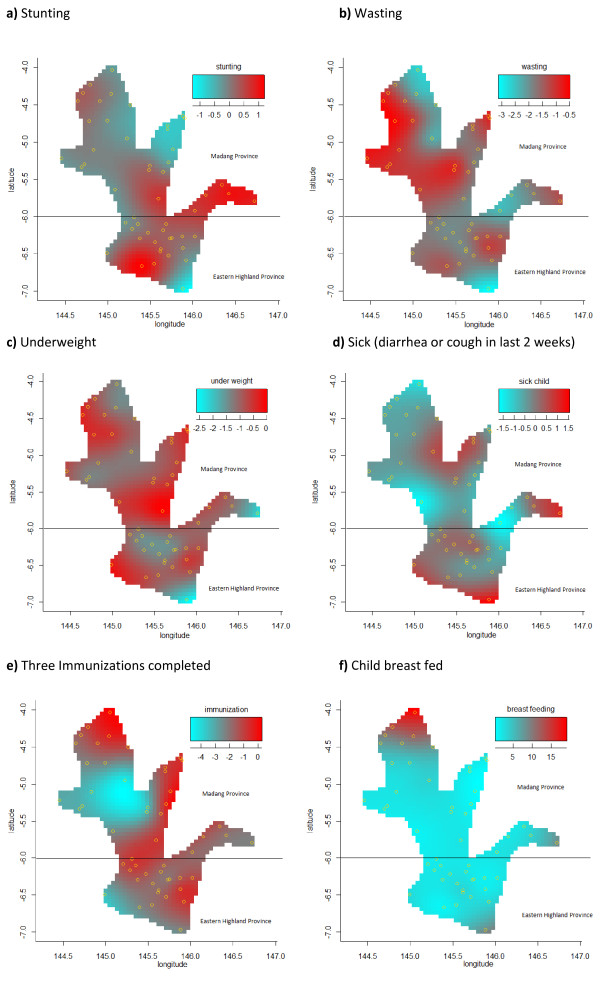
**Estimated pixel-size in figures is 1.88. a)** Stunting, **b)** Wasting, **c)** Underweight, **d)** Sick (diarrhea or cough in last 2 weeks), **e)** Three Immunizations completed, **f)** Child breast fed.

We created plots over the study region to test the null hypothesis that the outcome of interests does not depend on location (i.e. flat surface—blue). Figures 2a-2f show the image plot of age adjusted estimated bivariate geographical variation at districts level for the log odds of stunting, wasting, underweight, sickness, vaccine completion and breast feeding rates.

Visual inspection of Figure 2a indicates considerable spatial variation of stunting not just at province levels (upper and lower panels of Figure 2a) but at small-district levels as well (estimated degrees of freedom 19.3). According to this image, certain districts in both provinces had “hot-spots” i.e. excess prevalence of stunting compare to the neighbouring areas. However, this variation was not evident from the results presented in Table 1 which indicates that the prevalence of stunting being significantly higher among children who live in Eastern Highlands Province compared to those live in the Madang Province (59% vs. 49%, *P* = 0.019).

Figure 2b presents high prevalence areas for wasting among children at province as well as district levels. Consistent with the results given in Table 1, wasting was more common among children who lived in the Madang province (upper panel) compared to children who lived in the Eastern Highlands Province (lower panel). However, there was a considerable district level variation within each province as well.

Spatial variations of prevalence of underweight children were presented in Figure 2c. Results indicated considerable convexity (red areas) and concavity (blue areas) with suggesting a geographical diversity across the region at district-level rather than Province level with estimated degrees freedom 19.8. This result was consistent with Table 1 which indicates relatively high and similar prevalences of underweight children in both provinces (29% and 33%, *P* = 0.236).

Figure 2d presents the spatial variations of child being sick in the last 2 weeks, district level “hot-spots” were apparent in Madang (upper panel) and Eastern Highlands (lower panel) with estimated degrees of freedom of 17.4. In Table 1, prevalence of sick children were determined to be significantly higher In the Madang Province compared to the Eastern Highlands (49% versus 39%, *P* = 0.015). However, the image plot of this association suggests for certain “hot-spots” where the odds of child being sick significantly higher when compared to the neighbouring areas in both provinces—indicating great district level variations.

Although, vaccine completion rates were not statistically significant between the Eastern Highlands and Madang (Table 1: 21% versus 22% respectively, *P* = 0.650), results from a geoadditive model analysis revealed important district level features of the data with regard to the vaccine completion rates. According to the image plot presented in Figure 2e, The Western part of the Madang Province (upper—left panel) had the lowest rates of the vaccination completion rates (blue).

Figure 2f presents the spatial variations of breast feeding across the two provinces. Despite few district-specific relatively higher (red) and lower (blue) prevalences within each province, breast feeding was uniformly distributed across the provinces with estimated degrees of freedom of 3.

**Figure 3:**
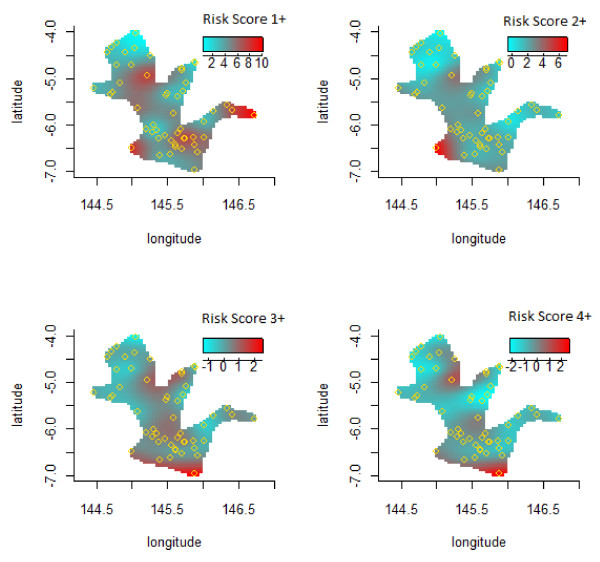
Geographic variations of risk score.

Figure 3 shows image plots of the age adjusted total risk score for 1 or more points (upper-left panel), 2 or more points (upper-right panel), 3 or more points (lower-left panel), 4 or more points (lower-right panel) which were calculated as the sum of the six modifiable risk factors selected in this study as described in the Methodology section. Collectively, these risk factors showed spatial variations across the targeted region. However, residents of the Eastern Highlands Province had more of the risk factors compared to the Madang Province.

### Child’s and caregiver’s age on stunting and wasting

**Figure 4:**
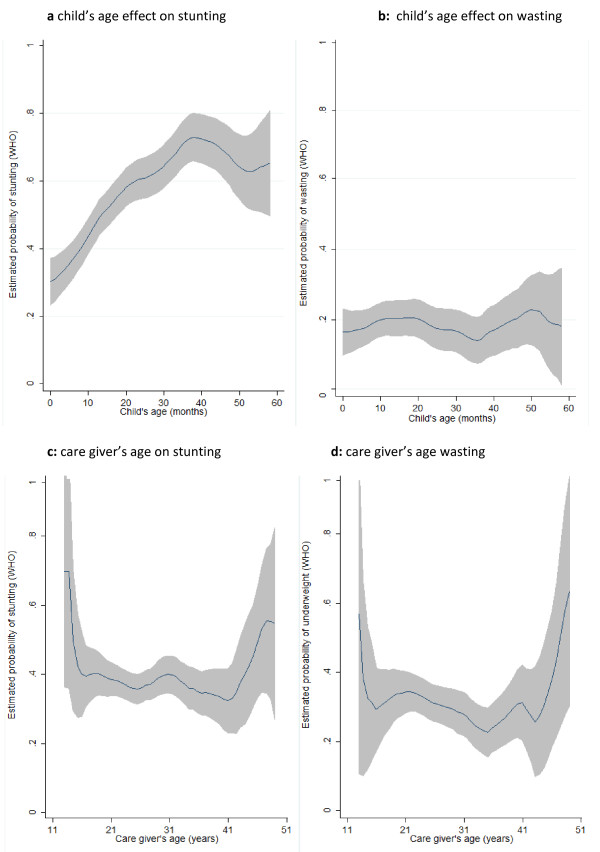
a: Child’s age effect on stunting, b: child’s age effect on wasting, c: care giver’s age on stunting, d: care giver’s age wasting.

Figures 4a and 4b show the flexible nonparametric modelling of the effect of the child’s age on the estimated prevalences of stunting and wasting respectively. We observed influence of age on the prevalence of stunting would be in the form of inverse U-shape, with the highest prevalence observed among children aged between 20 to 40 months (Figure 3a). This looks quite reasonable as younger children are likely to be breast fed and therefore they would be protective against stunting. Despite a slight upward trend in prevalence after age 40 months, there was no notable association between the prevalence of wasting and the child’s age.

Figures 4c and 4d show the flexible nonparametric modelling of the effect of the caregiver’s age on the estimated prevalences of stunting and wasting among children respectively. We see that the influence of caregiver’s age on the prevalence of stunting and wasting takes a U-shaped form, with the highest prevalences were associated with youngest and oldest age groups of the caregivers.

## Discussion

Based on the literature, we have a good understanding of the reasons and severe consequences of the childhood malnutrition [11,12]. However, these characteristics are not uniformly distributed across any population and may partially explain the varied etiology of that characterizes children in PNG. Therefore, evaluating the impacts of interventions may be difficult because communities may vary in their responses to an intervention and it may not be possible to make the best analytical adjustments for features associated with this variability. Effective strategies to prevent childhood malnutrition are likely to be those that address as many of the local factors as possible.

The objective of this paper was to describe the state of geographical variations of severe malnutrition such as stunting and wasting among a cohort of children who participated the study from Eastern Highlands and Madang Provinces in PNG. Our data clearly indicates one of the highest rates of stunting among children under the age of 5 in world. According to our knowledge, Eastern Highland Province had one of the highest rates of stunting reported in last 20 years with 59%. This prevalence was even higher than the one observed among children (aged <5 years) in Guatemala in 1995 (56%) [13]. Beside this unacceptably high childhood malnutrition figures, malaria, tuberculosis, typhoid, and pneumonia remain the largest causes of death amongst adults in PNG. This country is also responsible for the majority of new HIV infections across the entire South Pacific region [14-16].

By identifying high-risk areas of malnutrition, the current study made it possible to stratify district-specific risk spatially. Although, the entire country has been known to have one of the world’s highest rates of child death rates because of the various reasons such as malaria, measles, meningitis and malnutrition, identification of the hot-spots demonstrates the high geographical variability of stunting and wasting over the targeted region. The use of geographical information of the children’s residents made it possible to analyse these variations precisely, at the level of small-districts, improving our knowledge of the malnutrition in two provinces of PNG.

Because of the strong geographical variation, comparing the health outcomes at provincial levels do mask important district features and may even lead to misleading conclusions. This can be easily observed comparing the results between Table 1 and the statistical image plots presented in Figures 2a-2 f. It is evident that spatial effects of districts within the same province can vary a lot. Therefore, the interpretations for regions drawn from Table 1 will be biased for some of the districts within a region.

The result of the nonlinear effect of child’s age on stunting suggests that children aged 20–40 months old are the most vulnerable group—particularly compared to those younger than 20 months. Although this result contradicts some of the literature which links the malnutrition of young children to insufficient breast milk in early years, this could influence child’s health differently in the PNG setting where poverty is a part of life [17].

Our study also showed increasing trends in stunting and wasting when child was cared by young and old caregivers. Studies found strong link between the caregiver’s socioeconomic characteristics and the child’s malnutrition status. This is also consistent with the literature, children become more vulnerable when they are cared for by very young (such as older siblings) or very aged (relatives) [18,19].

It is clear that preventing or treating growth retardation in countries with poor economic conditions may play a key role in social and economic development. Therefore, there is an urgent need for monitoring of the nutritional status in developing countries such as PNG. Children require routine care to ensure that they are not being subjected to excessive periods of under-nutrition which can play a major role in promoting future productivity and overall social and economic development.

Previous studies linked many variables to stunting and wasting without accounting for geographical effects [1,3]. Describing geographical variations of malnutrition among children is important for scaling-up interventions. Although these data can provide snapshots of the malnutrition status and its determinants at local community level, further analyses and population-based data sources are needed to obtain a more complete picture of malnutrition in this region.

Our study has several limitations. First, we were not able to determine wealth and employment of the household. Studies reported that the poor socioeconomic status of a household had an association to stunting in children in developing countries [20,21]. Second, the original birth weight of all the children was not known which could be a crucial factor in determining from the start the growth pattern of the child, but also more importantly the deficits in the child’s growth [22]. Third, other anthropometric measurements that are now included in the WHO Growth Standard namely: head circumference-for-age (0–60 months), arm circumference-for-age, triceps skin-for-age, subscapular skinfold-for-age (3–60 months) were not available in our study. The use of these other anthropometric measurements in determining the nutritional status of children is a crucial aspect in the monitoring of child development [23]. The physical development of the children’s children physical is also being is also correlated to their interaction with their environment, how they move about to gain food for instance. Therefore, it is also important to assess a child’s overall development according to a child’s motor development. Fourth, current study only collected information (such as age and education level) from the caregivers rather than parents (particularly mothers). However, in PNG, mothers are usually the immediate and first care giver of the child, is an important attribute of improving and maintaining the nutrition of the child [24,25]. Therefore, our results regarding the characteristics of the caregivers can be broadly related to the mother’s characteristics of the child. In addition, mother’s malnutrition status was not collected. Maternal health studies have showed that if the mother was malnourished she would most likely give birth to low birth weight babies [26]. Consequently, babies would not fully develop physically and psychologically. Finally, the data used for this analysis were from 2003–4. However, more recent literature [27] suggest that child-mortality and stunting rates remain unacceptably high.

Despite its limitations, investigating small-scale geographical disparities using appropriate statistical approaches such as the one used in this study are useful to describe the features of the sparsely populated large geographical areas in detail.

These results may not be surprising since all the previous nutritional studies in PNG have reported low dietary intakes of protein and, in most cases of energy [28,29]. Papua New Guinea has also been classified by the World Health Organisation as an area where clinical vitamin A deficiency (VAD) exists [30]. For example, according to the National Health Survey/National Nutritional Survey (NHS/NNS) which was conducted in 1982/83, approximately 90% of the population was estimated to have had poor nutritional status [31]. In a study conducted among a cohort of children who lived in the East Sepik Province (north of Madang), protein and zinc deficiency were evident and significantly associated with stunting [32]. Low energy and protein levels were also reported among children who lived in Lufa (Eastern Highlands Province) and Kaul (Madang Province) [33]. Data on the status about iodine nutrition in children in Papua New Guinea (PNG) are scarce. However, at least one published literature indicated moderate iodine deficiency among a cohort of 350 school children (6–12 years old) in the Southern Highlands Province [34].

## Conclusions

Current study reports unacceptably high rates of prevalence of stunting and wasting among children younger than 5 years of age in PNG. Efforts to reduce the prevalence of stunting and wasting should focus on providing humanitarian and technical assistance to improve both dietary intake and overall health of children.

## Competing interests

The authors declare that they have no competing interests.

## Authors’ contribution

HW analyzed the data. PS was the principal investigator of the study; NL and IS implemented the study. HW, NL, IS and PS interpreted the results and drafted the manuscript. All authors read and approved the final manuscript.
